# Treatment of rheumatoid arthritis with baricitinib or upadacitinib is associated with reduced scaffold protein NEDD9 levels in CD4+ T cells

**DOI:** 10.14814/phy2.15829

**Published:** 2023-09-28

**Authors:** Viktoria Golumba‐Nagy, Shuaifeng Yan, Eva Steinbach‐Knödgen, Jan Thiele, Ruth L. Esser, Thomas H. Haak, Anastasia Nikiforov, Anja Meyer, Tamina Seeger‐Nukpezah, David M. Kofler

**Affiliations:** ^1^ Laboratory of Molecular Immunology, Division of Rheumatology and Clinical Immunology, Department I of Internal Medicine Faculty of Medicine and University Hospital Cologne, University of Cologne Cologne Germany; ^2^ University of Applied Sciences Utrecht Utrecht The Netherlands; ^3^ Center for Integrated Oncology Aachen Bonn Cologne Duesseldorf Cologne Germany

**Keywords:** baricitinib, CD4+ T cells, NEDD9, rheumatoid arthritis, tofacitinib, upadacitinib

## Abstract

The JAK/STAT pathway plays a crucial role in the pathogenesis of rheumatoid arthritis (RA) and JAK inhibitors have emerged as a new group of effective drugs for RA treatment. Recently, high STAT3 levels have been associated with the upregulation of the scaffold protein NEDD9, which is a regulator of T‐cell trafficking and promotes collagen‐induced arthritis (CIA). In this study, we aimed to reveal how treatment with JAK inhibitors affects NEDD9 in CD4+ T cells from RA patients. We analyzed NEDD9 expression in CD4+ T cells from 50 patients treated with either baricitinib, tofacitinib, or upadacitinib and performed cell migration assays to assess the potential influence of JAK inhibitor treatment on CD4+ T‐cell migration. We observed that treatment with baricitinib and upadacitinib is associated with reduced NEDD9 expression in CD4+ T cells. In contrast, NEDD9 levels were not altered during treatment with tofacitinib. Moreover, treatment with baricitinib was associated with a significantly reduced migratory capacity of effector CD4+ T cells but not with impaired migration of Treg cells. This study reveals previously unknown associations between JAK inhibitor treatment and NEDD9 expression and indicates that JAK inhibitors could reduce effector T‐cell migration.

## 1 INTRODUCTION

Rheumatoid arthritis (RA) is a common autoimmune disease characterized by infiltration of leukocytes into inflamed joints. B cells, CD4+, and CD8+ T cells, macrophages, neutrophiles, and other immune cells are implicated in the complex pathogenesis of the disease (Radu & Bungau, 2021). Pathogenic CD4+ T cells in the synovial tissue of patients with RA have pro‐inflammatory properties and their frequency correlates with disease activity (Floudas et al., 2022). In recent years, Janus kinase (JAK) inhibitors have emerged as a group of new effective drugs for the treatment of RA. JAK inhibitors attenuate inflammation by inhibiting JAK/STAT pathways involved in cytokine signaling. The JAK inhibitors baricitinib, tofacitinib, and upadacitinib inhibit JAK1 with varying selectivity for other JAK isoforms (Traves et al., 2021). While these JAK inhibitors have similar efficacy in patients with RA, they modulate distinct cytokine pathways to varying degrees and are associated with different rates of reported adverse events (AE) (McInnes et al., 2019; Traves et al., 2021). A better understanding of potential off‐target effects could help to reduce side effects and to increase the efficiency of JAK inhibitors.

The scaffold protein NEDD9 (neural precursor cell expressed, developmentally downregulated 9; also known as Cas‐L or HEF1) is a critical mediator of T‐cell trafficking (Ohashi et al., 1999; Regelmann et al., 2006; van Seventer et al., 2001). NEDD9 promotes chemokine‐induced T‐cell migration and regulates homing to lymph nodes (Gu et al., 2012; Huang et al., 2015). In addition, NEDD9 drives T‐cell infiltration into inflamed joints of mice with experimental autoimmune arthritis (Miyake‐Nishijima et al., 2003). Immunohistochemical analysis of joints from mice with autoimmune arthritis revealed a high number of NEDD9+ lymphocytes in the synovium (Miyake‐Nishijima et al., 2003). Moreover, knockout of NEDD9 attenuates collagen type II‐induced arthritis (CIA), indicating a protective role for NEDD9 deficiency in arthritis development (Katayose et al., 2015). Interestingly, NEDD9 can be significantly induced in tumor cells with high STAT3 levels, but not in tumor cells with low STAT3 levels (Tan et al., 2019). To reveal a potential link between the JAK/STAT pathway and NEDD9 in CD4+ T cells, we analyzed potential associations between JAK inhibitor treatment and NEDD9 expression levels in CD4+ T cells from RA patients.

## 2 MATERIALS AND METHODS

### 2.1 Blood samples

Peripheral blood was drawn from 50 RA patients treated with either baricitinib (4 mg QD), tofacitinib (5 mg BID), or upadacitinib (15 mg QD) in the outpatient clinic of the University Hospital Cologne. All patients fulfilled the 2010 ACR/EULAR classification criteria. Patients treated with JAK inhibitors received treatment for at least 6 months at the time point when blood was drawn. In accordance with the Declaration of Helsinki, written informed consent was obtained from the patients before blood was drawn. This study was approved by the Ethics Committee of the University Hospital Cologne (no. 13‐091). Age‐ and sex‐matched healthy individuals and RA patients without current treatment served as controls. The patients' characteristics are summarized in Table 1.

**TABLE 1:** Patients’ characteristics.

	Age	Female (%)	Disease duration (years)	DAS28‐CRP	RF+	ACPA+	CRP	ESR
RA bari (*n* = 26)	61.3 ± 7.8	15 (57.7%)	9.3 ± 5.1	2.25 ± 1.7	72%	67%	4.5 ± 3.6	19.0 ± 6.9
RA tofa (*n* = 13)	45.8 ± 6.2	10 (76.9%)	10.7 ± 8.6	2.0 ± 1.9	70%	66%	3.1 ± 2.8	17.5 ± 8.2
RA upa (*n* = 11)	57.5 ± 9.4	8 (72.7%)	11.2 ± 7.5	1.9 ± 1.5	65%	64%	4.7 ± 4.9	18.8 ± 7.6
RA w/o (*n* = 12)	53.1 ± 6.7	9 (75%)	3.6 ± 4.9	3.8 ± 2.1	68%	67%	14.9 ± 4.2	28.1 ± 9.2
HC (*n* = 15)	47.3 ± 4.1	10 (66.7%)	n/a	n/a	n/a	n/a	n/a	n/a

Abbreviations: RA, rheumatoid arthritis; bari, baricitinib 4 mg QD; tofa, tofacitinib 5 mg BID, upa, upadacitinib 15 mg QD; w/o, without treatment for ≥6 weeks or newly diagnosed; HC, healthy control; DAS28‐CRP, disease activity score of 28 joints based on CRP; RF, rheumatoid factor; ACPA, anticitrullinated protein; CRP, c‐reactive protein; ESR, erythrocyte sedimentation rate; n.a., not applicable.

*Note*: Values are shown in mean ± SEM.

### 2.2 CD4+ T‐cell isolation

Peripheral blood mononuclear cells (PBMC) were isolated using density gradient centrifugation as described previously (Pan Biotech) (Bund et al., 2006; Gloyer et al., 2022; Wendtner et al., 2004). CD4+ T cells were isolated by negative selection using MACS T‐cell isolation kit (Cat. No. 130‐096‐533, Miltenyi Biotech). The purity of the isolated cells was verified by flow cytometry and was at least 96%. Cell numbers and cell viability were assessed using the CellCountess (Life Technologies GmbH).

### 2.3 Western blot analysis

Purified human CD4+ T cells from RA patients were lysed with cell lysis buffer (BioLegend Inc.). The detection of protein concentration was conducted with the BCA Protein Assay Kit (Cell Signaling Technology®). For gel running, 4%–15% gradient polyacrylamide gels were used (Bio‐Rad Laboratories). Blotting was performed with the TransBlot® Turbo™ Transfer System (Bio‐Rad Laboratories) by using the PVDF membrane. The following antibodies were used for the protein detection: anti‐HEF1/NEDD9 (2G9) mouse mAb (Cat. No. 4044), anti‐mouse IgG HRP‐linked antibody (Cat. No. 7076), and anti‐GAPDH (D16H11) XP® rabbit mAb (HRP conjugate) (Cat. No. 8884) (all from Cell Signaling Technology®). Detection was performed by using ChemiDoc™ MP Imaging System (Bio‐Rad Laboratories), and data were analyzed by the ImageJ software (NIH). PC9 wt cells served as a positive control and PC9^nedd9−/nedd9–^ KO cells as a negative control for NEDD9 expression. PC9 cells are derived from human adenocarcinoma from lung tissue. The cells were kindly provided by Dr. Tamina Seeger‐Nukpezah (University of Cologne).

### 2.4 Gene ontology (GO)

Differentially expressed genes (DEGs) were defined as those with fold change >2 and *p* value < 0.01. DEGs were visualized as a heatmap using TBTools (Yan et al., 2021) (GSE106911, obtained from Gene Expression Omnibus database).

### 2.5 Flow cytometry

Flow cytometry was performed on the Gallios 10/3 flow cytometer and the results were analyzed by Kaluza Analysis Software (Both from Beckman Coulter). Preparation of cells was performed as described previously (Bruck et al., 2022; Klasen et al., 2019; Kofler et al., 2006). In brief, cells were stained with cell surface targets including CD4 (Cat. No. 347413), CD25 (Cat. No. 340907), and CD127 (Cat. No. 566399), then fixed and permeabilized by the BD Cytofix/Cytoperm Kit (BD Bioscience) or the True‐NuclearTM Transcription Factor Buffer Set (BioLegend Inc.) according to the manufacturer's instructions. Anti‐FoxP3 antibodies (Cat. No. 320116) and isotype antibodies (Cat. No. 981812) were purchased from Bio Legend Inc. Dead cells were excluded by the LIVE/DEADTM Fixable Dead Cell Stain Kit (Invitrogen, Thermo Fisher Scientific). Treg cells were defined as CD4^+^CD25^high^CD127^low^FoxP3^+^ T cells.

### 2.6 T‐cell transwell migration assays

The migratory capacity of CD4+ effector T cells and Treg cells was evaluated by chemo‐attractant transwell migration assay as described previously (Meyer et al., 2021; Yan et al., 2021). In brief, 6.5 mm transwells with 5 μm pore size (CorningTM Incorporated Costar) were equilibrated for 2 h in ex vivo 15 media (Lonza) supplied with 1% human serum and 1% penicillin–streptomycin (both Sigma–Aldrich) (Yan et al., 2021). In the next step, 1 × 10^6^ CD4+ T cells were seeded in transwell plates and incubated for 4 h under cell culture conditions (Yan et al., 2021). 50 ng/mL CCL20 (Cat. No. 583802, BioLegend Inc.) was used as a chemo‐attractant in the lower compartment (Yan et al., 2021). Where indicated, CD4+ T cells from untreated RA patients or from healthy controls were incubation in vitro with tofacitinib or baricitinib (Cat. No. S5001 and S5754, respectively, Selleck Chemicals) for 24 h prior to the migration assay. The total number of migrated CD4+ effector T cells or Treg cells in the lower chamber was counted by hemo‐cytometer and Treg cells were identified using antihuman FoxP3, CD25, and CD127 antibodies (all BioLegend Inc.) (Yan et al., 2021).

### 2.7 Statistical analysis

Statistical analysis was performed using SPSS®. Data were analyzed using one‐way ANOVA and Tukey's multiple comparison test. Data are presented as mean ± SEM (*p < 0.05, **p < 0.01, ***p < 0.001, ****p < 0.0001).

## 3 RESULTS

### 3.1 NEDD9 expression is suppressed in CD4+ T cells from patients treated with baricitinib or upadacitinib

**FIGURE 1:**
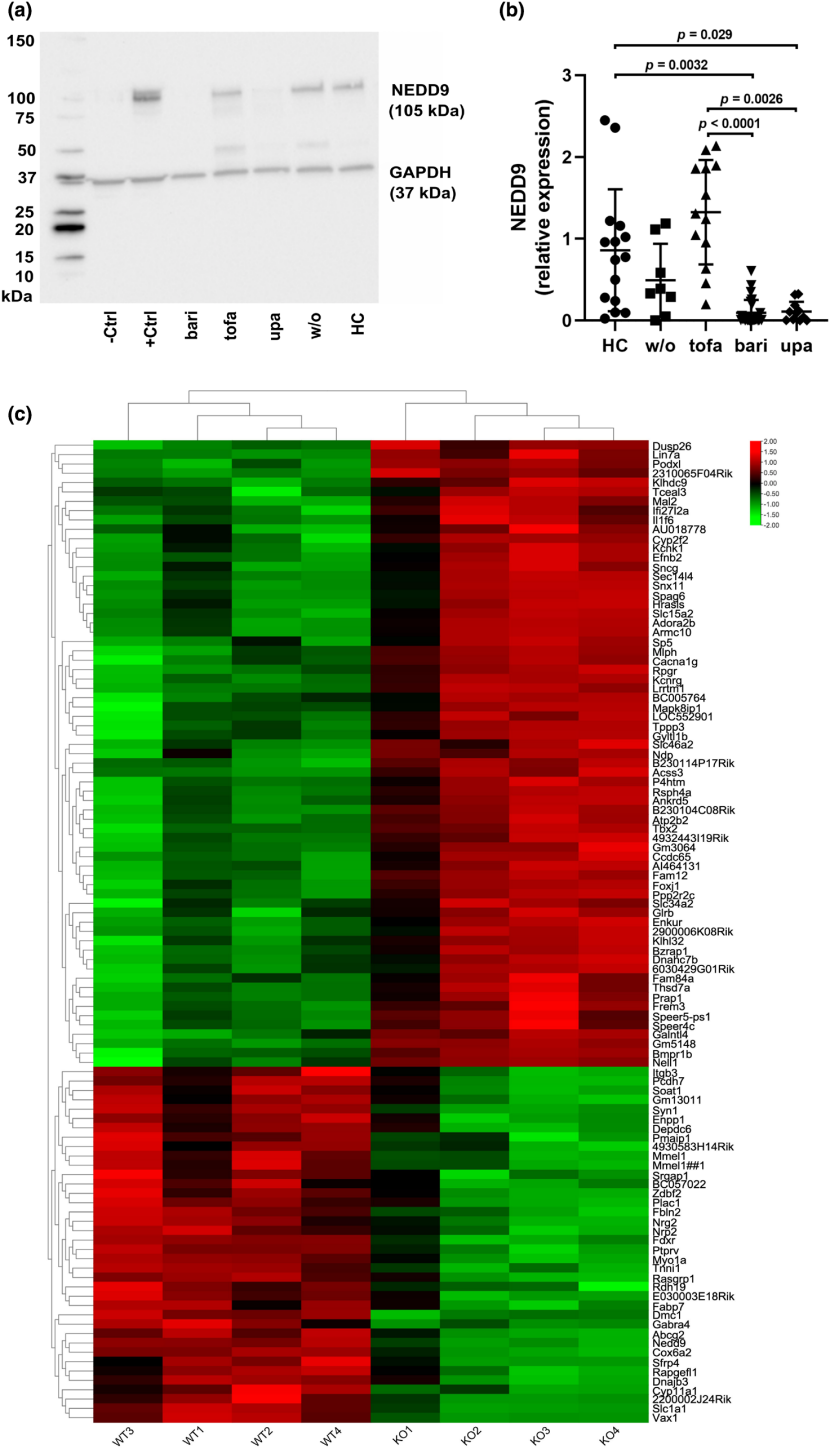
Baricitinib and upadacitinib are associated with suppressed NEDD9 levels in ex vivo CD4+ T cells. (a) Representative example of western blot analysis of NEDD9 in ex vivo CD4+ T cells from the peripheral blood of RA patients and healthy controls (HC). (b) NEDD9 expression in CD4+ T cells analyzed by western blot (HC, *n* = 15; w/o, *n* = 8; tofa, *n* = 13; bari, *n* = 26; upa, *n* = 11). (c) Heatmap shows differentially expressed genes in ovarian tumor cells from Nedd9^−/−^ and wild‐type mice (GSE106911, obtained from Gene Expression Omnibus database, |FC| > 2 and *p* < 0.01) (Gabbasov et al., 2018). –ctrl, negative control; +ctrl, positive control; HC, healthy controls; w/o, RA patients without current treatment; tofa, tofacitinib; bari, baricitinib; upa, upadacitinib. Data are presented as mean ± SD. Significance was calculated using one‐way ANOVA and Tukey's multiple comparison test.

We assessed the ex vivo expression level of NEDD9 by western blot in CD4+ T cells from 50 patients treated with JAK inhibitors. The patients' characteristics are summarized in Table 1. Our analysis revealed that treatment with baricitinib is associated with significantly reduced NEDD9 levels in CD4+ T cells as compared to CD4+ T cells from patients without treatment (relative expression 0.09 ± 0.03 vs. 0.63 ± 0.12, *p* = 0.0057). Similar results were observed in CD4+ T cells from patients treated with upadacitinib (0.11 ± 0.04 vs. 0.63 ± 0.12, *p* = 0.0398), while NEDD9 expression was not decreased in CD4+ T cells from patients receiving tofacitinib (0.98 ± 0.14 vs. 0.63 ± 0.12, *p* = 0.2661) (Figure 1a, b). NEDD9 expression levels in CD4+ T cells were similar between healthy individuals, untreated RA patients, and patients treated with tofacitinib.

### 3.2 NEDD9 knockout is associated with downregulation of genes that are implicated in cell migration

In a next step, we aimed to verify if loss of NEDD9 is accompanied by induction of compensatory mechanisms, which could replace the role of NEDD9 in cell migration. Therefore, we compared gene expression profiles of NEDD9‐negative and NEDD9‐positive cells using data sets of gene analysis of ovarian tumor cells from NEDD9 knockout mice and wilde‐type mice. Interestingly, we observed a significant downregulation of various genes involved in cell migration, including *PCDH7*, *SOAT1*, *NRG2*, and *FABP7*, in cells from NEDD9 knockout mice (Figure 1c). In addition, cells from NEDD9 knockout mice exhibited increased levels of *Dusp26*, a phosphatase associated with decreased cell migration.

### 3.3 Baricitinib treatment is associated with reduced migratory capacity of effector CD4+ T cells

**FIGURE 2:**
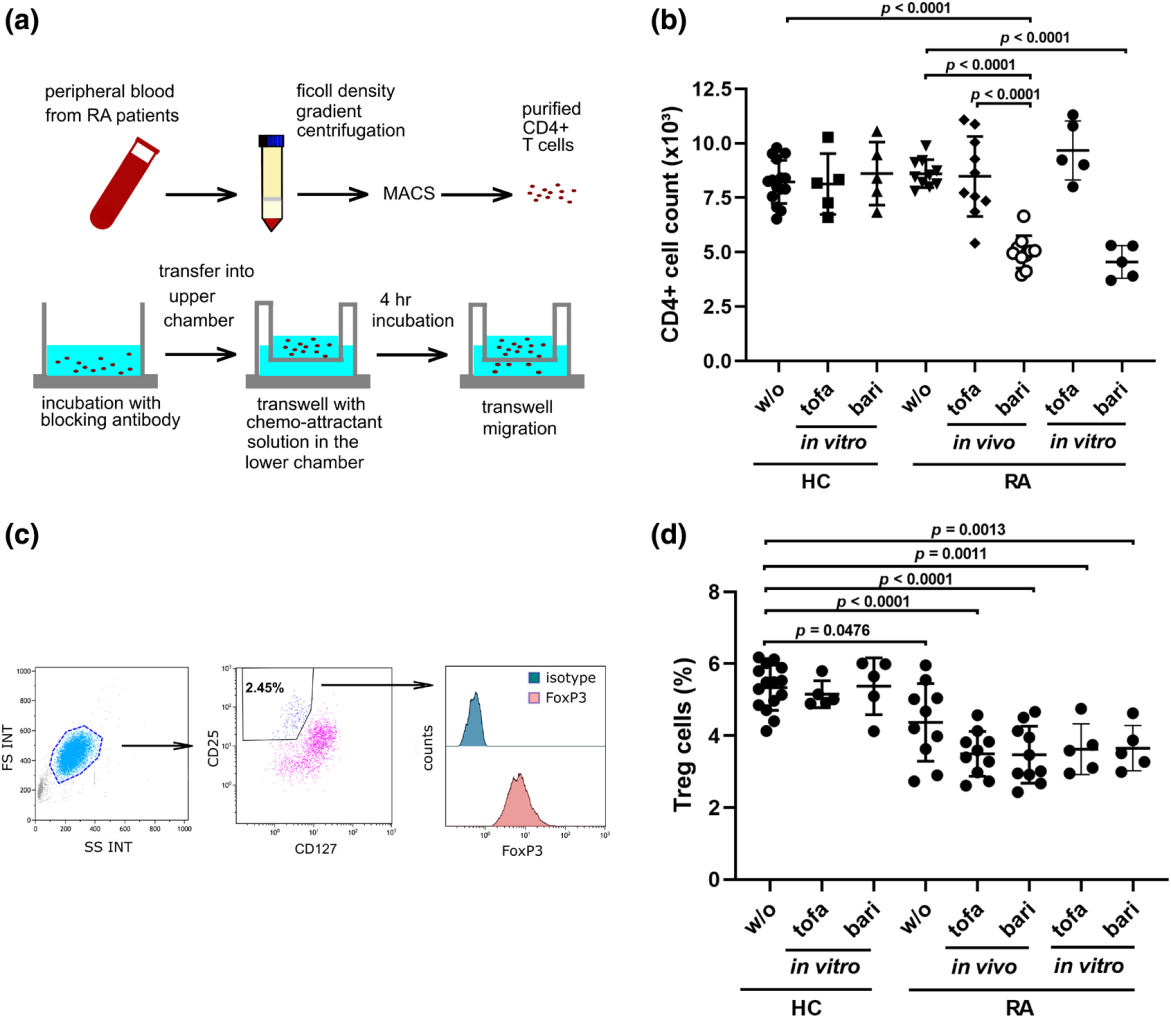
Baricitinib is associated with reduced in vitro migratory capacity of effector CD4+ T cells. (a) In vitro migration assay; schematic view of experimental setup. (b) In vitro migration of CD4+ T cells from RA patients (HC: w/o, *n* = 15; tofa [in vitro], *n* = 5; bari [in vitro], *n* = 5; RA: w/o, tofa [in vivo] and bari [in vivo], *n* = 10; RA: tofa [in vitro] and bari [in vitro], *n* = 5). Where indicated, CD4+ T cells from untreated RA patients or from healthy controls were incubated in vitro with tofacitinib or baricitinib for 24 h prior to the migration assay. (c) Gating strategy for the identification of FoxP3^+^CD127^low^CD25^high^ Treg cells. (d) In vitro Treg cell migration. HC, healthy controls; w/o, RA patients without current treatment; tofa, tofacitinib; bari, baricitinib. Data are presented as mean ± SD. Significance was calculated using one‐way ANOVA and Tukey's multiple comparison test.

As NEDD9 is a regulator of leukocyte migration, we verified if NEDD9 deficient CD4+ T cells from patients treated with baricitinib migrate less than CD4+ T cells from patients treated with tofacitinib in an in vitro migration assay (Figure 2a). CD4+ T cells from patients treated with either tofacitinib or baricitinib were seeded in transwell plates and were allowed to migrate toward CCL‐20 for 4 h under cell culture conditions. CCL20 was used to study chemotaxis because the CCL20 receptor CCR6 is highly expressed on various CD4+ T‐cell subsets, including Treg cells, Th17 cells, and Th22 cells. We observed a significantly reduced ability of CD4+ T cells from patients with baricitinib treatment to migrate toward chemokines compared with CD4+ T cells from patients with tofacitinib treatment (migrated cells = 5.0 ± 0.2 × 10^3^ vs. 8.5 ± 0.6 × 10^3^, *p* < 0.0001) (Figure 2b). Importantly, in vitro cultivation of CD4+ T cells from untreated RA patients or healthy individuals with tofacitinib or baricitinib showed similar results compared with CD4+ T cells from patients treated with these JAK inhibitors.

### 3.4 Baricitinib treatment is not associated with altered Treg cell migration

Interestingly, the percentage of CD127^low^CD25^high^FoxP3^high^ Treg cells among migrated CD4+ T cells was similar between patients treated with tofacitinib and patients treated with baricitinib (3.5 ± 0.2% vs. 3.5 ± 0.3%, *p* = 0.9999) (Figure 2c,d). The percentage of Treg cells was slightly lower in untreated RA patients compared with healthy controls (4.4 ± 0.3% vs. 5.4 ± 0.2%; *p* = 0.0476). Moreover, the percentage of migrated Treg cells was neither significantly altered in the tofacitinib group nor in the baricitinib group compared with untreated patients (3.5 ± 0.2% and 3.5 ± 0.3%, respectively).

## 4 DISCUSSION

Baricitinib is a potent JAK1 and JAK2 inhibitor. In addition, it shows moderate activity against TYK2 and very low activity against JAK3. In contrast, tofacitinib inhibits mainly JAK1 and JAK3 with less activity against TYK2 and JAK2. Upadacitinib is reported as a selective JAK1 inhibitor. In our study, we observed a significant suppression of NEDD9 in CD4+ T cells from RA patients treated with baricitinib or upadacitinib while tofacitinib had no influence on NEDD9 expression. This study is the first to report a link between JAK inhibitor treatment, NEDD9 expression, and CD4+ T‐cell migration. However, the design of our study does not allow any conclusion as to why baricitinib treatment is associated with reduced NEDD9 levels in CD4+ T cells and with diminished migratory capacity of CD4+ T cells. A possible explanation for these observations is that JAK1 inhibition might downregulate NEDD9 and JAK3 inhibition could abrogate this effect. Another explanation is that NEDD9 suppression could be mediated through unspecific off‐target effects of baricitinib and upadacitinib on other kinases, which are implicated in the regulation of NEDD9. Indeed, baricitinib has been shown to inhibit *focal adhesion kinase* (FAK), which is a strong promotor of NEDD9 induction (Karonitsch et al., 2018). Finally, the optimal dosage of tofacitinib for NEDD9 suppression was may not be reached in our patients, who received 5 mg BID. Further research is needed to explore the link between JAK inhibitors and NEDD9 suppression.

Recently, Gabbasov et al. have shown that NEDD9 deficiency is associated with reduced expression and activation of STAT3 (Gabbasov et al., 2018). STAT3 has various functions and is implicated in cell migration (Debidda et al., 2005; Hofmann & Kirsch, 2012). Therefore, it is possible that JAK2 inhibition reduces cell migration of CD4+ T cells by lowering the expression of NEDD9 and subsequently decreasing the expression and activation of STAT3. The possible link between NEDD9 and STAT3 regarding CD4+ T‐cell migration needs to be further evaluated.

The limit of this study is that our analysis does not allow any comparison of NEDD9 levels in a single person at different time points and with different JAK inhibitors. Instead, we compared different NEDD9 levels between subgroups of patients receiving either tofacitinib, baricitinib, or upadacitinib. Moreover, it remains unclear if the observed differences between JAK inhibitors have any clinical relevance. We used CCL20 to induce migration in our in vitro cell migration assays because the CCL20 receptor CCR6 is highly expressed on Treg cells, Th17 cells, Th22 cells, and on a small percentage of follicular T helper (Tfh) cells. Growing evidence suggests that the CCL20‐CCR6 axis is involved in the pathogenesis of RA (Fennen et al., 2021; Rodgers et al., 2020). In addition, increased plasma levels of CCL20 are found in the peripheral blood of RA patients and are positively corelated with DAS‐28, rheumatoid factor, and anti‐CCP antibodies (Pournazari et al., 2022). We observed lower frequencies of Treg cells among migrated CD4+ T cells from untreated RA patients as well as from patients treated with either tofacitinib or baricitinib. These findings are in line with previous reports showing that Treg cell frequencies are not significantly altered by treatment with JAK inhibitors (Meyer et al., 2019). Our data indicate that reduced migration of effector CD4+ T cells could add to the therapeutic effect of baricitinib.

## 5 CONCLUSION

This study reveals previously unknown associations between JAK inhibitor treatment and NEDD9 expression and indicates that JAK inhibitors could reduce effector T‐cell migration. Further research is required to determine whether these observations are linked together or if they are two independent phenomena. Moreover, the underlying mechanisms by which JAK inhibitors might regulate NEDD9 levels need to be revealed. Finally, future studies will help to clarify if NEDD9 expression could be a potential surrogate marker that correlates with the efficiency of baricitinib or upadacitinib in patients with RA.

## AUTHOR CONTRIBUTIONS

Viktoria Golumba‐Nagy, Shuaifeng Yan, Eva Steinbach‐Knödgen, Jan Thiele, Ruth L. Esser, Anastasia Nikiforov, and Thomas H. Haak performed the experiments. Viktoria Golumba‐Nagy, Shuaifeng Yan, Tamina Seeger‐Nukpezah, and David M. Kofler analyzed the data. David M. Kofler drafted the manuscript. All authors read and approved the final version of the manuscript.

## FUNDING INFORMATION

This work was supported by grants from Fritz Thyssen Foundation (10.17.2.019MN to DMK) and the Faculty of Medicine of the University of Cologne (grant 369/2020 to DMK).

## CONFLICT OF INTEREST STATEMENT

All authors declare that they have no competing interests regarding the publication of this article.

## ETHICS STATEMENT

This study was approved by the Ethics Committee of the University Hospital Cologne (no. 13‐091) and was performed in accordance with the Declaration of Helsinki.

## Data Availability

All data used to support the findings of this study are available from the corresponding author upon reasonable request.

## References

[phy215829-bib-0001] Bruck, C. , Golumba‐Nagy, V. , Yan, S. , Esser, R. L. , Thiele, J. , Stahl, D. , Pesch, C. T. , Steinbach‐Knodgen, E. , & Kofler, D. M. (2022). Th1 and Th17 cells are resistant towards T cell activation‐induced downregulation of CD6. Clinical Immunology, 238, 109025. 35487454 10.1016/j.clim.2022.109025

[phy215829-bib-0002] Bund, D. , Mayr, C. , Kofler, D. M. , Hallek, M. , & Wendtner, C. M. (2006). Human Ly9 (CD229) as novel tumor‐associated antigen (TAA) in chronic lymphocytic leukemia (B‐CLL) recognized by autologous CD8+ T cells. Experimental Hematology, 34(7), 860–869. 16797413 10.1016/j.exphem.2006.04.010

[phy215829-bib-0003] Debidda, M. , Wang, L. , Zang, H. , Poli, V. , & Zheng, Y. (2005). A role of STAT3 in rho GTPase‐regulated cell migration and proliferation. The Journal of Biological Chemistry, 280(17), 17275–17285. 15705584 10.1074/jbc.M413187200

[phy215829-bib-0004] Fennen, M. , Weinhage, T. , Kracke, V. , Intemann, J. , Varga, G. , Wehmeyer, C. , Foell, D. , Korb‐Pap, A. , Pap, T. , & Dankbar, B. (2021). A myostatin‐CCL20‐CCR6 axis regulates Th17 cell recruitment to inflamed joints in experimental arthritis. Scientific Reports, 11(1), 14145. 34239010 10.1038/s41598-021-93599-6 PMC8266846

[phy215829-bib-0005] Floudas, A. , Neto, N. , Orr, C. , Canavan, M. , Gallagher, P. , Hurson, C. , Monaghan, M. G. , Nagpar, S. , Mullan, R. H. , Veale, D. J. , & Fearon, U. (2022). Loss of balance between protective and pro‐inflammatory synovial tissue T‐cell polyfunctionality predates clinical onset of rheumatoid arthritis. Annals of the Rheumatic Diseases, 81(2), 193–205. 34598926 10.1136/annrheumdis-2021-220458

[phy215829-bib-0006] Gabbasov, R. , Xiao, F. , Howe, C. G. , Bickel, L. E. , O'Brien, S. W. , Benrubi, D. , do, T. V. , Zhou, Y. , Nicolas, E. , Cai, K. Q. , Litwin, S. , Seo, S. , Golemis, E. A. , & Connolly, D. C. (2018). NEDD9 promotes oncogenic signaling, a stem/mesenchymal gene signature, and aggressive ovarian cancer growth in mice. Oncogene, 37(35), 4854–4870. 29773902 10.1038/s41388-018-0296-y PMC6119087

[phy215829-bib-0007] Gloyer, L. , Golumba‐Nagy, V. , Meyer, A. , Yan, S. , Schiller, J. , Breuninger, M. , Jochimsen, D. , & Kofler, D. M. (2022). Adenosine receptor A2a blockade by caffeine increases IFN‐gamma production in Th1 cells from patients with rheumatoid arthritis. Scandinavian Journal of Rheumatology, 51, 1–5. 35023427 10.1080/03009742.2021.1995956

[phy215829-bib-0008] Gu, J. J. , Lavau, C. P. , Pugacheva, E. , Soderblom, E. J. , Moseley, M. A. , & Pendergast, A. M. (2012). Abl family kinases modulate T cell‐mediated inflammation and chemokine‐induced migration through the adaptor HEF1 and the GTPase Rap1. Science Signaling, 5(233), ra51. 22810897 10.1126/scisignal.2002632 PMC3602906

[phy215829-bib-0009] Hofmann, H. D. , & Kirsch, M. (2012). JAK2‐STAT3 signaling: A novel function and a novel mechanism. JAKSTAT, 1(3), 191–193. 24058769 10.4161/jkst.20446 PMC3670243

[phy215829-bib-0010] Huang, Y. , Clarke, F. , Karimi, M. , Roy, N. H. , Williamson, E. K. , Okumura, M. , Mochizuki, K. , Chen, E. J. H. , Park, T. J. , Debes, G. F. , Zhang, Y. , Curran, T. , Kambayashi, T. , & Burkhardt, J. K. (2015). CRK proteins selectively regulate T cell migration into inflamed tissues. The Journal of Clinical Investigation, 125(3), 1019–1032. 25621495 10.1172/JCI77278 PMC4362242

[phy215829-bib-0012] Karonitsch, T. , Beckmann, D. , Dalwigk, K. , Niederreiter, B. , Studenic, P. , Byrne, R. A. , Holinka, J. , Sevelda, F. , Korb‐Pap, A. , Steiner, G. , Smolen, J. S. , Pap, T. , & Kiener, H. P. (2018). Targeted inhibition of Janus kinases abates interfon gamma‐induced invasive behaviour of fibroblast‐like synoviocytes. Rheumatology (Oxford), 57(3), 572–577. 29228301 10.1093/rheumatology/kex426

[phy215829-bib-0013] Katayose, T. , Iwata, S. , Oyaizu, N. , Hosono, O. , Yamada, T. , Dang, N. H. , Hatano, R. , Tanaka, H. , Ohnuma, K. , & Morimoto, C. (2015). The role of Cas‐L/NEDD9 as a regulator of collagen‐induced arthritis in a murine model. Biochemical and Biophysical Research Communications, 460(4), 1069–1075. 25847598 10.1016/j.bbrc.2015.03.156

[phy215829-bib-0014] Klasen, C. , Meyer, A. , Wittekind, P. S. , Waqué, I. , Nabhani, S. , & Kofler, D. M. (2019). Prostaglandin receptor EP4 expression by Th17 cells is associated with high disease activity in ankylosing spondylitis. Arthritis Research & Therapy, 21(1), 159. 31253169 10.1186/s13075-019-1948-1 PMC6599260

[phy215829-bib-0015] Kofler, D. M. , Mayr, C. , & Wendtner, C. M. (2006). Current status of immunotherapy in B cell malignancies. Current Drug Targets, 7(10), 1371–1374. 17073599 10.2174/138945006778559120

[phy215829-bib-0016] McInnes, I. B. , Byers, N. L. , Higgs, R. E. , Lee, J. , Macias, W. L. , Na, S. , Ortmann, R. A. , Rocha, G. , Rooney, T. P. , Wehrman, T. , Zhang, X. , Zuckerman, S. H. , & Taylor, P. C. (2019). Comparison of baricitinib, upadacitinib, and tofacitinib mediated regulation of cytokine signaling in human leukocyte subpopulations. Arthritis Research & Therapy, 21(1), 183. 31375130 10.1186/s13075-019-1964-1 PMC6679539

[phy215829-bib-0017] Meyer, A. , Wittekind, P. S. , Kotschenreuther, K. , Schiller, J. , von Tresckow, J. , Haak, T. H. , & Kofler, D. M. (2019). Regulatory T cell frequencies in patients with rheumatoid arthritis are increased by conventional and biological DMARDs but not by JAK inhibitors. Annals of the Rheumatic Diseases, 80, e196. 31744827 10.1136/annrheumdis-2019-216576

[phy215829-bib-0018] Meyer, A. , Yan, S. , Golumba‐Nagy, V. , Esser, R. L. , Barbarino, V. , Blakemore, S. J. , Rusyn, L. , Nikiforov, A. , Seeger‐Nukpezah, T. , Grüll, H. , Pallasch, C. P. , & Kofler, D. M. (2021). Kinase activity profiling reveals contribution of G‐protein signaling modulator 2 deficiency to impaired regulatory T cell migration in rheumatoid arthritis. Journal of Autoimmunity, 124, 102726. 34555678 10.1016/j.jaut.2021.102726

[phy215829-bib-0019] Miyake‐Nishijima, R. , Iwata, S. , Saijo, S. , Kobayashi, H. , Kobayashi, S. , Souta‐Kuribara, A. , Hosono, O. , Kawasaki, H. , Tanaka, H. , Ikeda, E. , Okada, Y. , Iwakura, Y. , & Morimoto, C. (2003). Role of Crk‐associated substrate lymphocyte type in the pathophysiology of rheumatoid arthritis in tax transgenic mice and in humans. Arthritis and Rheumatism, 48(7), 1890–1900. 12847683 10.1002/art.11047

[phy215829-bib-0020] Ohashi, Y. , Iwata, S. , Kamiguchi, K. , & Morimoto, C. (1999). Tyrosine phosphorylation of Crk‐associated substrate lymphocyte‐type is a critical element in TCR‐ and beta 1 integrin‐induced T lymphocyte migration. Journal of Immunology, 163(7), 3727–3734. 10490968

[phy215829-bib-0021] Pournazari, M. , Feizollahi, P. , Roghani, S. A. , Assar, S. , Soufivand, P. , Soleymani, B. , Bahrehmand, F. , Kish, Z. M. , & Taghadosi, M. (2022). Increased plasma levels of CCL20 in peripheral blood of rheumatoid arthritis patients and its association with clinical and laboratory parameters. Clinical Rheumatology, 41(1), 265–270. 34477989 10.1007/s10067-021-05899-x

[phy215829-bib-0022] Radu, A. F. , & Bungau, S. G. (2021). Management of Rheumatoid Arthritis: An overview. Cell, 10(11), 1–33. 10.3390/cells10112857 PMC8616326 34831081

[phy215829-bib-0023] Regelmann, A. G. , Danzl, N. M. , Wanjalla, C. , & Alexandropoulos, K. (2006). The hematopoietic isoform of Cas‐Hef1‐associated signal transducer regulates chemokine‐induced inside‐out signaling and T cell trafficking. Immunity, 25(6), 907–918. 17174122 10.1016/j.immuni.2006.09.014

[phy215829-bib-0024] Rodgers, L. C. , Cole, J. , Rattigan, K. M. , Barrett, M. P. , Kurian, N. , McInnes, I. B. , & Goodyear, C. S. (2020). The rheumatoid synovial environment alters fatty acid metabolism in human monocytes and enhances CCL20 secretion. Rheumatology (Oxford), 59(4), 869–878. 31497857 10.1093/rheumatology/kez378

[phy215829-bib-0025] Tan, M. S. Y. , Sandanaraj, E. , Chong, Y. K. , Lim, S. W. , Koh, L. W. H. , Ng, W. H. , Tan, N. S. , Tan, P. , Ang, B. T. , & Tang, C. (2019). A STAT3‐based gene signature stratifies glioma patients for targeted therapy. Nature Communications, 10(1), 3601. 10.1038/s41467-019-11614-x PMC6689009 31399589

[phy215829-bib-0026] Traves, P. G. , Murray, B. , Campigotto, F. , Galien, R. , Meng, A. , & Di Paolo, J. A. (2021). JAK selectivity and the implications for clinical inhibition of pharmacodynamic cytokine signalling by filgotinib, upadacitinib, tofacitinib and baricitinib. Annals of the Rheumatic Diseases, 80(7), 865–875. 33741556 10.1136/annrheumdis-2020-219012 PMC8237188

[phy215829-bib-0027] van Seventer, G. A. , Salmen, H. J. , Law, S. F. , O'Neil, G. M. , Mullen, M. M. , Franz, A. M. , Kanner, S. B. , Golemis, E. A. , & van Seventer, J. M. (2001). Focal adhesion kinase regulates beta1 integrin‐dependent T cell migration through an HEF1 effector pathway. European Journal of Immunology, 31(5), 1417–1427. 11465098 10.1002/1521-4141(200105)31:5<1417::AID-IMMU1417>3.0.CO;2-C

[phy215829-bib-0028] Wendtner, C. M. , Kofler, D. M. , Mayr, C. , Bund, D. , & Hallek, M. (2004). The potential of gene transfer into primary B‐CLL cells using recombinant virus vectors. Leukemia & Lymphoma, 45(5), 897–904. 15291346 10.1080/10428190310001638896

[phy215829-bib-0029] Yan, S. , Golumba‐Nagy, V. , Kotschenreuther, K. , Thiele, J. , Refaian, N. , Shuya, D. , Gloyer, L. , Dittrich‐Salamon, M. , Meyer, A. , Heindl, L. M. , & Kofler, D. M. (2021). Membrane‐bound IL‐6R is upregulated on Th17 cells and inhibits Treg cell migration by regulating post‐translational modification of VASP in autoimmune arthritis. Cellular and Molecular Life Sciences, 79(1), 3. 34913099 10.1007/s00018-021-04076-2 PMC8674172

